# Inequalities in child immunization coverage in Ghana: evidence from a decomposition analysis

**DOI:** 10.1186/s13561-018-0193-7

**Published:** 2018-04-11

**Authors:** Derek Asuman, Charles Godfred Ackah, Ulrika Enemark

**Affiliations:** 1 Institute of Statistical, Social and Economic Research, University of Ghana, E.N. Omaboe Building, P. O. Box LG 74, Legon, Ghana; 2 Section for Health Promotion and Health Services Research, Department of Public Health, Aarhus University, Bartholins Alle 2, 8000 Aarhus C, Denmark

**Keywords:** Immunization coverage, Childhood, Vaccination, Health inequality, Decomposition, Rural-urban, Ghana

## Abstract

Childhood vaccination has been promoted as a global intervention aimed at improving child survival and health, through the reduction of vaccine preventable deaths. However, there exist significant inequalities in achieving universal coverage of child vaccination among and within countries. In this paper, we examine rural-urban inequalities in child immunizations in Ghana. Using data from the recent two waves of the Ghana Demographic and Health Survey, we examine the probability that a child between 12 and 59 months receives the required vaccinations and proceed to decompose the sources of inequalities in the probability of full immunization between rural and urban areas. We find significant child-specific, maternal and household characteristics on a child’s immunization status. The results show that children in rural areas are more likely to complete the required vaccinations. The direction and sources of inequalities in child immunizations have changed between the two survey waves. We find a pro-urban advantage in 2008 arising from differences in observed characteristics whilst a pro-rural advantage emerges in 2014 dominated by the differences in coefficients. Health system development and campaign efforts have focused on rural areas. There is a need to also specifically target vulnerable children in urban areas, to maintain focus on women empowerment and pay attention to children from high socio-economic households in less favourable economic times.

## Background

Improving child health outcomes has been central to global development efforts over the last four decades. For example, the Millennium Development Goals (2000–2015) adopted by the United Nations in 2000 committed global agencies and national governments in Target 4 to reduce global child mortality rates. The succeeding Sustainable Development Goals (2016–2030) re-emphasizes the importance of improving child health and survival rates as an important global development objective. Such global commitments have led to increased public and private investments in promoting accessible and affordable child health intervention programs, particularly in nutrition and immunization services [18]. For example, GAVI has provided financial and technical support for the development of effective vaccines and low-cost immunization implementation programmes in developing countries [41].

The World Health Organisation (WHO) in 1974 launched the Expanded Programme on Immunization (EPI) as a public health initiative aimed at improving child health and survival through routine and universal immunization coverage. Generally, the EPI has been successful, increasing child immunization rates from 5% at the inception of the initiative to 83% in 2014 [14]. Despite these efforts, there exist substantial challenges to achieving universal coverage of child immunization in developing countries, especially within the Sub Saharan African (SSA) region [31]. The limited coverage of child immunisation programmes within the SSA region may attenuate the ability of countries in the region to improve child health outcomes [12]. For example, Liu et al. [31] estimates that SSA will account for 60% of global child deaths by 2030.

As such, there exist an urgent need to achieve universal coverage of full immunization as a mechanism to achieve the child mortality target of the SDGs. For policy purposes, it is imperative to examine and understand the factors that contribute to the utilization of immunization services in the sub-region for effective and targeted programme implementation. Achieving equity in immunization involves creating equal opportunities for all eligible children to have access to such services as well as identifying disadvantaged and vulnerable children who are at risk of being unvaccinated [29]. A number of socioeconomic and demographic factors have been identified to influence child immunization coverage. Evidence from earlier studies have shown child-specific, parental and household-level characteristics as important predictors of child immunization coverage.

At the child-level, there exist conflicting evidence of gender gaps in immunization coverage which may reflect cultural specific differences in the status of women. Studies such as, Corsi et al. [16] and Pal [37] report significant gender disparities in India, with girls disadvantaged. Antai [5] on the other hand finds that girls are more likely to receive full immunization in Nigeria whilst Tsawe et al. [42] and Landoh et al. [28] do not find significant gender differentials in immunization coverage in Swaziland and Togo respectively. In addition to gender, other child-specific characteristics found to influence immunization status are birth order or parity [5, 19], age [38], and place of delivery [13, 36].

For parental-specific characteristics, studies on child immunization have focused extensively on maternal characteristics. Particularly, educational attainment and literacy [6, 7, 19, 36], employment status [13, 43], age and age at birth [5, 36], media exposure [13, 42], marital status [28] and religion [28, 36] have been found to have strong relationships with the immunization status of a child. The presence of such strong maternal effects on child immunization coverage is a reflection of the traditional childcare responsibility of mothers in most developing countries, which dovetails to the policy recommendation that empowering women in the household decision-making process, such as child health, may be crucial to achieving universal immunization coverage among underserved populations [5, 44].

Two household-level characteristics have been examined extensively in earlier studies on inequalities in child health outcomes – residence of the household and socioeconomic status measured by wealth or assets. The effects of these characteristics on child immunization coverage are however ambiguous, reflecting large cross-country differences. For example, Bugvi et al. [13], Olorunsaiye & Degge, [36] and Singh and Parasuraman [40] find inequalities in immunization coverage disfavoring children residing in households with lower socioeconomic status. On the other hand, Barata et al. [9] finds lower immunization rates among children from households with high socioeconomic status in Brazil. These findings may reflect differential opportunity of time costs that mothers or caregivers face in making regular visits to immunization centres [38, 40].

Equally, a number of studies have reported significant rural-urban disparities in child immunization coverage across developing countries. In developing countries where spatial inequalities in availability and access to health facilities and information are pervasive, rural communities remain underserved in a number of essential services. A group of studies such as Abadura et al. [1], Antai [5] and Bugvi et al. [13] report rural disadvantage in child immunization in Ethiopia, Nigeria and Pakistan. A second strand of studies has on the other hand reported lower immunization rates among children in urban areas, especially slum and informal settlements in Kampala [8] and Nairobi [19]. Such differences may arise from differences in development strategies as well as the definition of rural and urban areas.

There exist significant differences across countries and localities in the sources of inequalities in child immunizations due to significant variations in structural, cultural and institutional settings [26]. The presence of such differences require that further studies are undertaken to identify the country-specific extent and sources of inequalities in child immunization coverage. In this paper, we seek to contribute to the literature by examining rural-urban inequalities in child immunization coverage in Ghana. The objectives of the paper are twofold. First, we examine the determinants of full immunization coverage of children aged 12–59 months using a logistic regression technique. Specifically, the paper assesses the probability that a child receives the basic WHO required immunizations. The paper then proceeds to decompose the difference in the probability of full immunization coverage between rural and urban areas into a part attributable to differences in observed characteristics and an unexplained component, which may reflect structural and institutional differences in health systems between urban and rural areas.

We employ data from the fifth and sixth waves of the Ghana Demographic and Health Survey (GDHS) conducted in 2008 and 2014 respectively. The healthcare delivery systems in Ghana witnessed substantial transformation between 2008 and 2014, especially in the expansion of primary healthcare facilities in rural areas and access to health insurance. The period also saw stable economic growth, made significant gains towards poverty reduction and a rapid rate of urbanization. Using data from the two waves of the survey enables us to compare the changes in the nature and sources of inequalities in child immunization coverage within the structural and demographic transformations over the period.

The rest of the paper is organised as follows, Section 2 provides an overview of the child immunization programme in Ghana. Section 3 focuses on the estimation approaches including the decomposition analysis. Section 4 discusses the data source, description of variables and summary statistics of the sample. Section 5 consists of two parts. The first section discusses the results from the determinants of full immunization status whilst the second section focuses on the results of the inequality decomposition. The paper ends with a summary of the main findings and policy recommendations based on the findings.

## Child immunization in Ghana

Ghana launched the Expanded Programme on Immunizations in 1978. The launch of the immunization programme was in response to a national strategy to reduce maternal and infant morbidity and mortality from vaccine preventable diseases. Six antigens were introduced at the inception of the immunization programme for expectant mothers and children. Child vaccinations included Bacillus Calmette-Guèrin (BCG) vaccination against tuberculosis; measles; Diphtheria-Pertusis-Tetanus (DPT) and oral polio for children under 12 months old and tetanus toxoid vaccination for pregnant women. In 1992, a yellow fever vaccination was added to the national immunization programme for children.

The National Immunization Programme has adopted the guidelines proposed by the WHO and UNICEF on child immunization. The guidelines recommend that a child receives one dose each of BCG and measles and three doses of DPT and polio. A dose of BCG vaccine is required at birth or at the first clinical contact. Equally, a dose of polio is recommended at birth or within 13 days of birth. The required doses of DPT and polio vaccines are recommended to be taken at 6, 10 and 14 weeks of age. The measles vaccines are required to be taken at 9 months of age. It is recommended that a child receives the basic required immunizations before 12 months of age.

The immunization programme in Ghana has been reviewed periodically since the inception of the programme. The DPT vaccine was replaced with a pentavalent vaccine (DPT-Hep B-Hib) in 2002 to include immunizations against Hepatitis B and *Haemophilus Influenza* type B. Two new vaccines – pneumococcal and rotavirus to protect children from pneumococcal diseases particularly pneumonia and diarrhoea respectively were introduced in 2012. A child is required to receive three doses of the pneumococcal vaccine at 6, 10 and 14 weeks of age and two doses of the rotavirus at 6 and 10 weeks old respectively. The measles only vaccine was replaced in 2013 with a measles-rubella vaccine also to be given at 9 months. A second dose of the measles-only vaccine was introduced in the same year to be given as a booster at 18 months.

## Methods

Traditionally, studies examining socioeconomic inequalities in health outcomes have applied the concentration index and concentration graph approaches [27, 45]. These techniques though useful for measuring the health inequalities, do not explain the pathways through which socioeconomic factors influence observed inequalities in health outcomes between groups. Wagstaff et al. [46] proposes a methodology to decompose the concentration index into a part that shows the contribution of each characteristic to the concentration index and a component that is residual, which is the part of inequality that cannot be explained by the variations in contributing factors across socioeconomic groups. However, the approach proposed by Wagstaff et al. [46] is appropriate for decomposing ranked-based socioeconomic inequalities. The technique, however, may not be useful for examining subgroup inequalities in health such as rural-urban differences, not based on socioeconomic rankings (see [35] for a discussion).

This paper adopts an alternative approach. We estimate a logit model to examine the determinants of immunization status and proceeds to decompose the observed rural-urban differentials in immunization coverage based on a non-linear decomposition technique. This section consists of two parts and discusses the methodological approaches adopted for the empirical estimations. The first part is focused on the econometric technique employed to examine the determinants of full immunization status among children in the sample. The second part presents the decomposition technique applied to assess the source and extent of rural-urban inequalities in Ghana.

### Determinants of child immunization status

The immunization status (*I*_*ijh*_) of child *i* of mother *j* in household *h* is modelled as a non-linear function of child-specific characteristics(*C*_*ijh*_), maternal characteristics (*M*_*jh*_), household socioeconomic characteristics (*Z*_*h*_) including region of residence, and an error term(*ɛ*_*ijh*_).

$$ {I}_{ijh}=F\left({C}_{ijh};{M}_{jh};{Z}_h;{\varepsilon}_{ijh}\right) $$

A reduced form model is estimated, where the dependent variable is binary and takes the value of 1 if the child is fully immunized and 0 if otherwise. The reduced form model is specified as

$$ {I}_{ijh}=\pi +{\alpha}_i{C}_{ijh}+{\beta}_i{M}_{jh}+{\delta}_i{Z}_h+{\varepsilon}_{ijh} $$

where *π* is a constant term and *α*, *β*, and *δ* are parameters to be estimated. The error term is random and assumed to be constant variance and mean of zero. For simplicity, we generalise the functional form of the reduced model to be estimated as

$$ I={X}^{\hbox{'}}\beta $$

such that *I* is the immunization status of the child, *X* is a vector of explanatory variables and *β* is a vector of coefficient to be estimated. The probability of a child being fully immunized conditioned on the independent variables is obtained as

$$ \Pr \left(I=1|X\right)=\Lambda \left({X}^{\hbox{'}}\beta \right)=\frac{e^{X^{\hbox{'}}\beta }}{1+{e}^{X\hbox{'}\beta }} $$

where л is the cumulative density function of the logistic distribution.

The coefficient estimated by the logit regression technique, similar to other limited dependent variable estimation models, only show the direction of the relationship between the covariates and the probability of a full immunization. In such models, the marginal effects represent the effect of a change in a covariate on the predicted probability of full immunization. Thus, marginal effects of the logit model are presented for ease of interpretation and discussion. The marginal effect with respect to a change in a covariate (*X*_*i*_) is computed as.

$$ \frac{\partial \Pr \left(I=1|X\right)}{\partial {X}_i}=\Lambda \left({X}^{\hbox{'}}\beta \right)\left[1-\Lambda \left({X}^{\hbox{'}}\beta \right)\right]{\beta}_i $$

Separate logit regressions are estimated for each survey round.

### Decomposition of inequalities in child immunization status

A common approach to examine differences in continuous outcomes between groups is the decomposition technique proposed by Oaxaca [34] and Blinder [10]. The Oaxaca-Blinder decomposition technique identifies two sources of outcome differentials between groups. The first component of the observed gap is the explained or endowment effect. The endowment effect captures differences in the outcome of interest that arises from observed differentials in the endowments or characteristics between the groups. The second components of sources of outcome differences is referred to as coefficient or return effect. The return effect is unexplained and is attributed to differences in the returns to endowments between groups. Thus, each group receives different returns for the same level of endowments. When applied to a labour market outcome such as wages, the returns effect has been attributed to discrimination against a disfavoured group. However, in the case of a health outcome, the returns effect may reflect the indirect effects of structural and institutional differences in health systems that affect the health seeking behaviours and attitudes differently between rural and urban areas [15].

The classical Oaxaca-Blinder decomposition technique has been extended to binary and other non-linear outcomes. Fairlie [20] and Fitzenberger, Kohn and Wang [22] have applied the extended technique to decompose differentials between groups when outcomes are binary dependent variables. The non-linear decomposition model assumes that the conditional expectation of the probability of the immunization status of a child is a non-linear function of a vector of characteristics. Following the generalized structure of the reduced form of the model, separate models are estimated for rural and urban areas as.

$$ {I}_r=\Lambda \left({X}_r^{\hbox{'}}{\beta}_r\right)\;\mathrm{for}\kern0.17em \mathrm{subsample}\kern0.17em \mathrm{of}\kern0.17em \mathrm{rural}\kern0.17em \mathrm{and} $$

$$ {I}_u=\Lambda \left({X}_u^{\hbox{'}}{\beta}_u\right)\;\mathrm{for}\kern0.17em \mathrm{the}\kern0.17em \mathrm{subsample}\kern0.17em \mathrm{of}\kern0.17em \mathrm{urban}\kern0.17em \mathrm{areas}. $$

To decompose the rural-urban inequalities in immunization status (Δ*I*), is rewritten as:

$$ \varDelta I={I}_r-{I}_u=\overline{\Lambda \left({X}_r^{\hbox{'}}{\beta}_r\right)}-\overline{\Lambda \left({X}_u^{\hbox{'}}{\beta}_u\right)} $$

Consider a counterfactual conditional probability of immunization status (I^*^) which evaluates the conditional probability of immunization status if the coefficients for rural and urban areas are the same.

$$ {I}^{\ast }=\overline{\Lambda \left({X}_r^{\hbox{'}}{\beta}_u\right)} $$

The decomposition of the gaps is obtained as

$$ \varDelta I={I}_r-{I}^{\ast }+{I}^{\ast }-{I}_u=\overline{\Lambda \left({X}_r^{\hbox{'}}{\beta}_r\right)}-\overline{\Lambda \left({X}_r^{\hbox{'}}{\beta}_u\right)}+\overline{\Lambda \left({X}_r^{\hbox{'}}{\beta}_u\right)}-\overline{\Lambda \left({X}_u^{\hbox{'}}{\beta}_u\right)} $$

The first term, $\overline{\Lambda \left({X}_r^{\hbox{'}}{\beta}_r\right)}-\overline{\Lambda \left({X}_r^{\hbox{'}}{\beta}_u\right)}$ measures the returns effect, whereas the second term $\overline{\Lambda \left({X}_r^{\hbox{'}}{\beta}_u\right)}-\overline{\Lambda \left({X}_u^{\hbox{'}}{\beta}_u\right)}$ represents endowment effect. The decomposition technique, in addition, assesses the contribution of each covariate to the rural-urban inequalities in immunization coverage in Ghana.

## Data source, description of variables and summary statistics

### Data source

The data used in this paper comes from the Ghana Demographic and Health Survey (GDHS), implemented by the Ghana Statistical Service (GSS), the Ghana Health Service (GHS) and the National Public Health Reference Laboratory of the GHS [24]. The primary objective of the GDHS is to generate reliable information on fertility, family planning, infant and child mortality, maternal and child health, and nutrition. In addition, the dataset contains information on the characteristics of the respondents and the household. To date, six rounds of the GDHS have been collected using similar procedures.

The surveys follow a two-stage sample design. The first stage involves selecting sample points or clusters, consisting of enumeration areas. The second stage involves a systematic listing of households in selected clusters. A pre-determined number of households are randomly selected from each cluster to constitute the total sample size of households. All women aged 15–49 years who are either permanent residents of the household or visitors who stayed in the household the night before the survey were eligible to be interviewed. The birth history of each woman was collected. For children under five years, the immunization status of each child was taken either from their vaccination or verbal recall from the mother. The working sample for this study is drawn from the children between 12 and 59 months old from the fifth and sixth waves of the survey collected in 2008 and 2014 respectively. The sample for the study includes 2147 children of 1781 mothers residing in 1700 households in 2008. The sample for 2014 on the other hand includes 4386 children of 3652 mothers in 3506 households.

### Description of variables

The dependent variable is a binary indicator of immunization status of a child. The variable captures whether or not a child had received required basic immunizations. The WHO and UNICEF recommends that a child receives one dose each of BCG and measles and three doses each of polio vaccines and DPT. Full immunization is restricted to the basic immunizations to enable comparison between the two survey rounds as well as with studies from other countries and settings.

The explanatory variables, as stated earlier, include child-specific, maternal and household characteristics. The choice of these variables was influenced by the earlier studies. Child-specific characteristics include age in months, sex, birth order and place of delivery. Child age in months has been categorised into four groups, 12–23 months, 24–35 months, 36–47 months and 48–59 months. The youngest category (12–23 months) is adopted as the referenced category to compare age-differentials in immunization coverage. Similarly, the sex of the child is included to examine the presence of gender differentials. The birth order of the child captures the attitudes of mothers towards immunization as the number of children increases. Some of the immunizations are recommended at birth or first clinical contact. As such, the place of delivery of the child is important to determining whether a child will receive the required immunizations.

The maternal characteristics are crucial to immunization status as the mothers are the primary caregivers within the Ghanaian society. The maternal characteristics included are the age at birth, years of completed schooling and marital status. Religious affiliation of the mother is included as an explanatory variable to examine religious differences in immunization status. Health authorities embark on media campaigns to educate mothers on the importance of child immunization as well as the schedule of immunization programmes. Thus, a mother’s exposure to media may be important to immunization coverage. A dummy of variable of media exposure is therefore included. A mother is defined to be exposed to the media if she has access to information through newspapers and magazines, radio and television. To capture other informal access to information on immunization, the number of women of reproductive age resident in the household is included as an additional variable.

Other maternal characteristics include ownership of a valid health insurance policy, which is adopted as a proxy to capture the health seeking behaviour of the mother. Membership of a health insurance has been found to increase demand for outpatient services through the provision of protection against the financial risk [2]. Mothers who make the effort to obtain a health insurance card, demonstrate, ceteris paribus, a stronger underlying preference for health and health care than others. This further materializes in higher use of health services among those insured. The nature of the economic activity of the mother is categorised into unemployed, self-employed and working for family or others and included as an explanatory variable. This variable is important as it may be adopted to capture time use and opportunity cost of waiting at immunization centres for mothers as well as the capacity to pay for other services such as transportation. Though child immunization services in Ghana are free, mothers may face significant costs to access health and immunization centres. Therefore, the ease of access to health facilities, captured by whether distance poses a challenge to women, is included as a dummy variable.

One of the objectives of this paper is to examine rural-urban inequalities in child immunization coverage. The main variable of interest is therefore the location of the household. A dummy variable that indicates whether a household is located in a rural or urban area is included to capture the rural-urban differentials. Household socioeconomic status is measured by a composite wealth index based on household ownership of selected assets, housing conditions and access to water and sanitation facilities. Three categories of household socioeconomic status are created – low, middle and high. The low category is adopted as the reference category to compare socioeconomic differences in immunization coverage. Lastly, regional dummies are included to capture variations in health attitudes and behaviours, social and cultural norms and belief and health systems across the ten administrative regions.

### Descriptive statistics

Summary statistics of the variables utilized for the empirical analysis is presented in Table 1. In both rounds of the survey, over two-thirds of the children aged 12–59 months had received the required basic immunizations. Females constitute about 48% of the sample in 2008 and 2014. The proportion of children delivered at health facilities increased from 57% in 2008 and 73%. Equally, the proportion of mothers with valid health insurance policies increased from 39% to 65% between 2008 and 2014. A free health insurance membership policy for pregnant women was initiated in 2008 as a mechanism to achieve the maternal and child health target of the MDG. The policy was aimed to improve access to prenatal, delivery and postnatal healthcare. This resulted in increased health insurance coverage for women and increased facility based delivery.

**Table 1:** Summary statistics of child-specific, maternal and household characteristics^a^

Characteristics	2008	2014
	Percentage	
Immunization coverage	71.7	71.4
Female child	48.4	48.3
Child delivered at health facility	56.7	73.0
Marital or consensual union	88.1	84.1
Distance to health facility is a problem	28.8	26.8
Has valid health insurance	39.1	65.2
Exposed to media	84.4	90.1
Rural	59.8	53.8
Age category of child		
12–23 months	26.1	26.1
24–35 months	23.5	25.6
36–47 months	24.0	24.8
48–59 months	26.5	23.5
Religious affiliation of mother		
No/Other religion	10.9	7.1
Christian	70.9	76.2
Moslem	18.2	16.7
Labour market status of mother		
Unemployed	9.5	15.7
Family/Other employee	18.6	20.6
Self-employed	71.9	63.7
Household wealth index		
Low	45.1	40.5
Middle	19.1	20.5
High	35.8	39.0
	Mean	
Mother’s age at birth	28.2	28.7
Mother’s years of completed schooling	5.8	6.0
Number of co-resident women	0.4	0.3
Number of observations	2147	4386

^a^Estimates adjusted for survey settings

The summary statistics shows significant changes in the nature of employment of mothers between 2008 and 2014. The proportion of mothers unemployed increased to nearly 16% in 2014 from 10% in 2008, whilst mothers in self-employment declined from 72% in 2008 to 64% in 2014. The shifts in employment status of mothers reflect the macroeconomic challenges such as a debilitating energy crisis that resulted in a decline in economic activity especially in the non-agricultural informal sector. The years of completed schooling for mothers remain low, at 6 years – corresponding to primary school completion in both 2008 and 2014.

The proportion of households in rural areas decreased from 60% in 2008 to 54% in 2014, a reflection of the rapid urbanisation witnessed in Ghana, a reflection of a demographic shift witnessed in Ghana as well as other countries within the SSA region. The summary statistics shows that the differences in household socioeconomic status in 2008 and 2014 rounds of the survey. 45% of households in 2008 had low socioeconomic status, whilst 36% fell within the high socioeconomic status category. In 2014, the proportion of households within the low socioeconomic category was 41% whilst 39% of households were high socioeconomic status. The shifts in the socioeconomic status of households between 2008 and 2014 is reflective of the relatively stable economic growth witnessed over the period and the resulting decrease in poverty in the country.

## Results and discussions

### Determinants of child immunization status

Table 2 presents the marginal effects of the logit regressions. The findings show the presence of significant rural-urban differences in the probability of a child being fully immunized in both survey rounds. Specifically, the results show that compared to children in urban areas, children residing in rural households are about 8% and 4% more likely to have received the basic required immunizations in 2008 and 2014 respectively. The finding departs from earlier studies in developing countries that report statistically significant rural disadvantage in child immunization coverage [1, 5, 13]. On the other hand, studies such as Awasthi et al. [7], Babirye et al. [8], and Egondi et al. [19] report that in spite of the physical access to health facilities and immunization centres in urban areas, there exist substantial barriers to immunization coverage in urban settings, with large underserved populations in slum and informal settings.

**Table 2:** Determinants of Full Childhood Immunizations, 2008–2014

	2008			2014		
VARIABLES	Full	Urban	Rural	Full	Urban	Rural
Rural	0.0770**			0.0448**		
	(0.0306)			(0.0191)		
23–35 months	−0.0137	0.0418	−0.0388	− 0.0207	− 0.0469	0.0039
	(0.0249)	(0.0414)	(0.0312)	(0.0177)	(0.0286)	(0.0224)
36–47 months	−0.1208***	− 0.0644	− 0.1489***	−0.0667***	− 0.0848***	−0.0516**
	(0.0261)	(0.0454)	(0.0323)	(0.0186)	(0.0303)	(0.0236)
48–59 months	− 0.1392***	− 0.1195***	− 0.1513***	− 0.1170***	− 0.1306***	− 0.1065***
	(0.0257)	(0.0457)	(0.0311)	(0.0191)	(0.0309)	(0.0244)
Female	−0.0081	−0.0060	− 0.0076	0.0049	0.0093	0.0037
	(0.0187)	(0.0315)	(0.0232)	(0.0132)	(0.0214)	(0.0168)
Birth order	−0.0056	− 0.0060	− 0.0028	− 0.0095*	−0.0067	− 0.0101
	(0.0072)	(0.0159)	(0.0082)	(0.0054)	(0.0094)	(0.0067)
Delivered at health facility	−0.0024	0.0423	−0.0136	0.0356**	0.0765**	0.0241
	(0.0225)	(0.0448)	(0.0263)	(0.0169)	(0.0379)	(0.0187)
Mother’s age at birth	0.0048**	0.0073*	0.0029	0.0056***	0.0070***	0.0045**
	(0.0022)	(0.0042)	(0.0027)	(0.0016)	(0.0026)	(0.0020)
Years of schooling	0.0038***	0.0041	0.0033**	0.0054***	0.0042	0.0056**
	(0.0015)	(0.0029)	(0.0016)	(0.0020)	(0.0030)	(0.0027)
Number of co-resident women	−0.0181	−0.0136	−0.0179	−0.0179*	−0.0344*	−0.0063
	(0.0131)	(0.0203)	(0.0169)	(0.0101)	(0.0179)	(0.0122)
Married/Consensual union	0.0254	0.0318	0.0181	0.0716***	0.0809**	0.0568**
	(0.0328)	(0.0534)	(0.0429)	(0.0215)	(0.0333)	(0.0282)
Christian	0.0741**	0.0833	0.0790**	0.0320	0.0266	0.0284
	(0.0315)	(0.0952)	(0.0351)	(0.0262)	(0.0567)	(0.0293)
Moslem	0.0806**	0.0610	0.0962**	0.0807***	0.0198	0.1044***
	(0.0349)	(0.1010)	(0.0402)	(0.0284)	(0.0605)	(0.0328)
Media exposure	0.0162	0.0265	0.0152	0.0303	0.0327	0.0274
	(0.0268)	(0.0663)	(0.0302)	(0.0214)	(0.0506)	(0.0232)
Employed – family/others	0.1169***	0.0449	0.1439***	−0.0097	−0.0338	0.0116
	(0.0386)	(0.0623)	(0.0502)	(0.0230)	(0.0356)	(0.0306)
Employed - self	0.1068***	0.0587	0.1228***	0.0306	0.0122	0.0493*
	(0.0349)	(0.0513)	(0.0469)	(0.0197)	(0.0299)	(0.0264)
Has valid health insurance	0.0585***	0.0955***	0.0449*	0.0458***	0.0617**	0.0343*
	(0.0210)	(0.0359)	(0.0265)	(0.0148)	(0.0249)	(0.0185)
Distance to facility is a problem	−0.0174	−0.0801*	−0.0037	−0.0213	−0.0289	− 0.0160
	(0.0212)	(0.0474)	(0.0242)	(0.0156)	(0.0290)	(0.0183)
Middle	0.0612**	0.0549	0.0600*	−0.0337	0.0003	−0.0410
	(0.0301)	(0.0662)	(0.0354)	(0.0208)	(0.0362)	(0.0267)
High	0.0926***	0.0638	0.1055**	−0.0878***	−0.0433	− 0.1313***
	(0.0328)	(0.0668)	(0.0424)	(0.0263)	(0.0377)	(0.0444)
Regional dummies	Yes	Yes	Yes	Yes	Yes	Yes
Observations	2147	709	1438	4386	1743	2643

Standard errors in parentheses ***p < 0.01, **p < 0.05, *p < 0.1

Demographic and health systems development may have contributed to the urban immunization disadvantage in Ghana. Over the last two decades, Ghana has witnessed a rapid pace of urbanisation, with substantial growth in slum and informal settlements. Thus, the growing number of underserved children in slum and informal settlements in urban areas in Ghana have contributed to the observed urban disadvantage in the coverage of child immunizations. Given the existence of large spatial disparities in access and utilization of health services and facilities, the child immunization strategy in Ghana appears to have place emphasis on rural areas, much to the neglect of underserved populations in urban areas. In line with this, there has been much emphasis on expanding basic health services in rural areas through the Community-based Health Planning and Services (CHPS) program. The urban-based CHPS has turned out to be much more challenging to implement as the population is more volatile and less well demarcated [4].

In terms of child-specific characteristics, there are no gender disparities in the immunization status of children in Ghana. Indeed, evidence of gender differences in child immunization status have largely been reported by studies from southern Asian countries where son preference are endemic [16, 37]. However, there exist statistically significant age differentials in the immunization status of children. Compared to children 12–23 months old, children between 36 and 47 months and 48–59 months olds are less likely to receive fully the basic immunizations. Age differentials in childhood immunization coverage have been reported in earlier studies. Such age disparities reflect the increasing trend in coverage with recent cohorts of children being fully immunized.

The results indicate a negative effect of the birth order of a child on the probability of a child receiving immunization in 2014. Adedokun et al. [1] and Antai [5] report that children of higher birth order are less likely to complete the required immunizations in Nigeria, and Corsi et al. [16] report same for India. The results suggest “*immunization fatigue*” of mothers as their interest to immunize their children as the number of children increases wanes [3]. Antai [5] on the other hand posits that the reduced likelihood of full immunization coverage of children of higher birth order reflects inter-sibling competition for parental care and limited household resources, leading to neglect.

There may be alternative explanations to the negative effect of child’s birth order on the probability of complete basic immunizations. First, the result may reflect the increased opportunity cost of time. The *indirect wage* of mothers in home production increases as the number of children increases. As such, as the high opportunity cost of time spent at a health centre to vaccinate a child of higher birth order reduces the probability of the child being fully immunized. Secondly, younger mothers may be better educated and informed on family planning and child health. The improved education thus reflects in fewer number of children and increased probability of complete immunization. Lastly, the results may be a reflection of a cohort effect – children with siblings have older mothers and the probability of taking children to vaccination decreases over time. The effect may be higher among old mothers than new mothers.

The place of delivery of a child shows a significant positive relationship with the probability of full immunization in 2014. The likelihood of full immunization is about 4% higher for children delivered in health facilities compared to children delivered at home. The importance of place of delivery to child immunization status is consistent to earlier studies such as Adedokun et al. [3] and Bugvi et al. [13]. Delivery at a health facility enables a child to receive the immunizations required at birth and provides an opportunity for the mother to be informed of the immediate schedules. The emergence of a significant relationship between the place of delivery of the child and probability of full immunization reflects the effects of improved access and utilisation of health services on child health. The reduction of the financial barriers to the demand for maternal and child health through the fee exemption policy under the National Health Insurance Scheme has contributed to achieving equity in child vaccinations in Ghana. Disaggregated by the place of residence, we find that the effect of the place of delivery is significant for urban areas only. This finding suggests the relative success of outreach programmes in rural areas where home vaccinations have been promoted through CHPS.

Religion is an important determinant of health seeking behaviours as well as health outcomes [28, 36]. The results indicate the presence of significant religious effects on the probability of full immunization status in 2008. Compared to mother with no or other religious backgrounds, children with Christian or Moslem mothers are more likely to be fully immunized. The effects of religion, however, appear to have been attenuated in 2014. The significant difference between the immunization of children born to Christian mothers and mothers with no and other religious affiliations disappears, whilst, Moslem mothers are more likely to complete the required immunization routine for their children.

The significant relationships between maternal characteristics and the probability of full childhood immunization indicate the importance of maternal characteristics to child immunization status. Similar to Adedokun et al. [3] and Antai [5], the results indicate a positive relationship between the age at birth of the mother and the probability of the child being fully immunized in 2008 and 2014. Given pervasive cultural disapproval of early childbirth, the results may reflect the attitude of health workers and community members towards teenage mothers leading such mothers to drop-out of the routine child immunization [5]. The effects of mother’s age at childbirth, thus, sheds light on the possible barriers that cultural values and belief systems impose on child immunization attitudes in Ghana. In addition, younger mothers have been found to have limited bargaining power in intra-household decision making, as young mothers may be required to seek the permission of other household members regarding decision concerning the health of the child [30, 39]. Thus, delays in household decision making may have negative consequences for child health outcomes.

Improving female and maternal education has been promoted globally as a mechanism to enhance child health outcomes, especially in developing countries. Indeed, there is a growing body of empirical literature that show significant effects of increased maternal education on improved child health outcomes [25, 32]. The results from the logit estimations are consistent with the findings of these previous studies such as Abadura et al. [1], Adedokun et al. [3] and Ataguba, Ojo and Ichoku [6]. An additional year of completed schooling of a mother exerts a positive effect of the probability of a child receiving the basic immunizations. Educational attainment of the mother enhances the access and reception of information as well as facilitates communication between the mother and health workers, leading to better understanding of immunization schedules and practices [28].

The labour market status of the mother is included as a proxy for the mothers’ time use as well as the opportunity cost of waiting at health and immunization centres. The variable may also capture the effect of the socioeconomic status of a woman on the immunization status of the child. Uthman et al. [43] finds that the probability that a child receives the full basic required immunizations is lower for children with unemployed mothers. Bugvi et al. [13] suggest that unemployed mothers and those in low paying occupations do not find the time and resources to travel to health centres for the immunization of their children, leading to incomplete immunization routines. On the other hand, employed mothers may face high opportunity cost of time foregone, thus causing them to forego routine visits to complete the basic immunization requirements. Our results in this paper are consistent with Uthman et al. [43]. The probability of full immunization coverage is higher for children with employed mothers in 2008. However, there exist no significant effect of maternal employment on immunization status of children in 2014 except for children in rural areas with self-employed mothers. The changes observed in the labour market status of mothers between 2008 and 2014 appear to have shifted the effects on the probability of full vaccination of children. An increase in unemployment increases the opportunity cost of foregone wages. As such, employed mothers faced higher opportunity cost for waiting time at vaccination centres in 2014. Other things equal, this would reduce the propensity of employed mothers to spend time at vaccination centres, thereby eroding the higher likelilhood of having their children fully immunized observed in 2008.

Enrolment in a health insurance scheme has been found to increase the utilisation of outpatient services and reduction of out-of-pocket expenditures [21]. Brugiavini and Pace [11] finds a positive effect of health insurance membership on maternal health in Ghana. Our results show that mothers with valid health insurance policy are more likely to complete the basic immunizations for their children. Thus, there exists a spill-over effect of mother’s attitude to their own health to the health outcomes of their children. Mothers with health insurance may generally seek care more often when ill or when their child is ill. Health workers may use this opportunity to catch up on missing immunizations, thus resulting in the higher coverage in this group. Equally, the positive relationship between health insurance policy and completed basic child immunization may reflect possible financial barriers to child immunization. Child immunizations are offered for free in Ghana. However, mothers without valid health insurance coverage may be deterred by the fear of financial payments for immunization services.

Previous studies have reported significant socioeconomic inequalities in the coverage of child immunization across developing countries. The socioeconomic status of the household is an indicator of standard of living and has been adopted as a proxy for opportunity cost of women’s time as well as financial access to healthcare. Ataguba, Ojo and Ichoku [6], Olorunsaiye and Degge, [36] and Singh and Parasuraman [40] report a disadvantage against children residing in households with low socioeconomic status. The present results from logit regressions reveal changes in the socioeconomic inequalities in child immunization coverage in Ghana between 2008 and 2014. Whilst children in high socioeconomic households are more likely to receive the full basic required immunizations compared to children from low socioeconomic households.

The results reveal a reversal of the disadvantage faced by households with low socioeconomic status in 2014 as there exist a significant negative effect of high household socioeconomic status and the probability of full childhood immunization. The presence of inequalities in child immunization against high socioeconomic households departs from a majority of studies on socioeconomic inequalities in child health outcomes. The results, however, reflect changing economic conditions witnessed between 2008 and 2014. A slowdown of economic activity exerted pressure on the time of women as such women may be required to engage in market activities to smoothen household standards of living. The increased demand for mother’s time in market activities may have led to an increase in the opportunity cost of time for mothers potentially leading mothers to pursue income-generating activities instead of completing the immunization routines for their children. Equally, the expansion of services and coverage through the CHPS initiative have also contributed to better access for least wealthy populations especially in rural areas.

### Decomposition of rural-urban inequalities in child immunization, 2008/2014

A summary of sources of rural-urban inequalities in child immunization coverage in Ghana in 2008 and 2014 obtained from the decomposition analysis are presented in Table 3. The rural subsample is the reference category for the decomposition of the gaps in immunization coverage. The findings reveal the existence of rural-urban disparities in the probability of a child receiving the basic immunizations. Unlike logit estimates, the decomposition analysis reveals that the direction of disparities in child immunization coverage changes between 2008 and 2014.

**Table 3:** Summary of Oaxaca-Blinder Decomposition Results

Sources	Overall	Overall
	2008	2014
Rural	0.6940***	0.7276***
	(0.0121)	(0.0086)
Urban	0.7348***	0.7011***
	(0.0165)	(0.0109)
Difference	−0.0408**	0.0265*
	(0.0205)	(0.0139)
Endowment / Explained	−0.1335***	−0.0196***
	(0.0286)	(0.0154)
Coefficient / Unexplained	0.0926**	0.0461**
	(0.0362)	(0.0204)
Coefficient / Unexplained (%)	41.0	70.2
Observations	2147	4386

Robust standard errors in parentheses ***p < 0.01, **p < 0.05, *p < 0.1

In 2008, the average probability of a child in a rural household to receive the full basic immunization is 0.6940 compared to an average probability of 0.735 for a child in an urban area. The rural-urban differential in the average probability of a children being fully immunized in 2008 is statistically significant at 5%. In terms of the source of the disparities in the probability of full immunization, the results further indicates that the endowment or explained effect contributes about 59% of the gap. Indeed, differences in the endowments or characteristics favours urban resident children. This suggests that on average, children residing in urban areas possess higher levels of endowments compared to their counterparts in rural areas. The coefficient or unexplained effect, however, favours children in rural areas. This result therefore, implies that the health system in Ghana, in terms of child immunization services reward rural households higher for the same level of characteristics compared to urban households. Given the disparities in access to health care services and facilities, the child immunization program in Ghana has adopted strategies that aim at reaching the most vulnerable households, especially in rural areas. This strategy, however, appear to neglect vulnerable children in fast growing slums and informal settlements in urban areas.

Table 4 shows the contribution of each group of characteristics to the rural-urban gap in child immunization coverage. The decomposition reveals that maternal and household characteristics are the significant contributors to the endowment effect. The locational differences in both group of characteristics contribute to widening the gap in the probability of a child being fully immunized between rural and urban areas.

**Table 4:** Sources of contributions to rural-urban inequalities in child immunization coverage

Characteristics	2008		2014	
	Endowment	Return	Endowment	Return
Child	−0.0075	−0.1042	−0.0260***	− 0.0254
	(0.0139)	(0.1060)	(0.0089)	(0.0564)
Maternal	−0.0379***	−0.1687	− 0.0398***	−0.0276
	(0.0117)	(0.2629)	(0.0101)	(0.1133)
Household	−0.0701***	0.0274	0.0602***	−0.0295
	(0.0262)	(0.0687)	(0.0231)	(0.0268)
Region dummies	−0.0180	− 0.0454	−0.0140**	− 0.1415**
	(0.0141)	(0.1416)	(0.0070)	(0.0701)
Constant		0.3835		0.2701**
		(0.2928)		(0.1339)
Observations	2147		4386	

Robust standard errors in parentheses ***p < 0.01, **p < 0.05, *p < 0.1

In 2014 however, the average probability of a child in a rural area to be immunized was 0.728 compared to 0.701 for a child in an urban area. The difference in the average probability of fully immunization between rural and urban areas is statistically significant at 10%. However, the coefficient or unexplained component of the gap dominates in 2014, accounting for 70.2% of the estimated rural-urban differentials in the probability of a full immunization of a child. The dominance of the coefficient effect may be attributed to the expansion of primary healthcare in rural Ghana between the periods though the CHPS programme The CHPS programme aims at improving access to primary healthcare and family planning services through community participation and mobilization. The number of functional CHPS facilities increased from 409 in 2008 to 2948 in 2014 [23]. The programme has achieved considerable success in rural areas whilst the implementation in urban areas has been challenging.

The endowment effects in 2014 arises from significant differences in child, maternal, household and regional characteristics. Child, maternal and regional differences favor children in urban households, thus, contributing to widening the gap in immunization coverage between rural and urban areas. Household characteristics, which includes the number of resident women of reproductive age and household socioeconomic status, contribute to the endowment effect in favour of rural-resident children. On the other hand, returns to region of residence – contribution to the unexplained or coefficient component – is positive in favour of children in urban areas. This implies that in each region, the health system rewards children in urban areas higher in terms of probability of full immunization than children in rural areas.

## Conclusion and policy recommendations

The objective of this paper was to investigate rural-urban inequalities in child immunization coverage in Ghana between 2008 and 2014. Improving child health outcomes constitute a primary development objective in developing countries, especially in SSA. Global development efforts over the last two decades – MDGs (2000–2015) and SDGs (2016–2030) - have emphasized the centrality of child health improvements to economic growth and development. Investments in early childhood health have been found to be essential to later-life outcomes such as cognitive ability, education, income and productivity [15]. Thus, socioeconomic disparities in health investments in early childhood may perpetuate intergenerational poverty and inequalities as well as deride the objective of equitable and inclusive growth.

The paper focuses on a sample of children between 12 and 59 months old, drawn from the two recent rounds of the Ghana Demographic and Health Survey conducted in 2008 and 2014. First, the paper examines the determinants of the probability of a child receiving the basic required immunizations of one dose each of BCG and measles and three doses each of polio vaccines and DPT using a logit regression technique. The second part of the paper decomposes the rural-urban inequalities in coverage of full child immunizations into a component attributed to differences in observed characteristics and a component arising from differences in returns to these characteristics using the Oaxaca-Blinder technique extended to non-linear outcomes.

The findings of the paper reveal significant rural-urban differentials in the probability of a child receiving the required immunization. Specifically, children in rural households are more likely to have completed the required immunizations compared to children in urban areas in both 2008 and 2014. In addition, child-specific, maternal and household characteristics exert significant effects on the probability of a child’s immunization status. The effects of maternal education and employment suggest the importance of women empowerment to child health outcomes in developing countries. The effect of household socioeconomic status on the probability of a child receiving the required immunizations changed between 2008 and 2014. The probability of full immunization was positive for children residing in households with high socioeconomic status in 2008. However, the effect of household socioeconomic status in 2014 shows that the probability of a child receiving the required immunizations is lower for children in households with high socioeconomic status.

The decomposition analysis of the rural-urban inequalities in child immunization coverage reveals the existence of significant disparities in the probability of a child receiving the full immunization. The direction of the disparities, however, differs in 2008 and 2014. In 2008, there exist a rural disadvantage in child immunization coverage. The gap in immunization coverage is dominated by endowment or explained effect. In 2014, there exist an urban disadvantage in child immunization. The emerging urban disadvantage may reflect the neglect of primary healthcare delivery in fast growing slums and informal settlements in urban areas. The coefficient or unexplained effect is the dominant source of the coverage gap in 2014, reflecting the concentration on immunization campaigns and improvement in access to primary healthcare in rural areas.

The findings of the paper provide insights for achieving universal child immunization coverage in Ghana. A number of policy recommendations may be drawn from the findings of this paper. First, and most importantly, policies aimed at reducing socioeconomic inequalities in child health in Ghana need to adjust focus and increasingly target vulnerable children in urban areas, particularly in slum and informal settlements. The rapid pace of urbanization in Ghana requires that public health policies incorporate strategies that address the demands of a growing urban population. Recent systematic reviews of the literature up to 2016 assessing interventions to improve routine immunization coverage in urban areas [33] and in low income urban or slum areas [17] identified only few studies in low and middle income countries, of which only 7 in total from SSA and only 4 of them less than ten years old. Both reviews point towards the need across low and middle income countries for further development and testing of interventions in view of the local context and with communities. National immunization campaigns should be targeted at mothers and children in slums and informal settlements in urban areas through community participation and mobilization as a means to increase coverage of immunization services in such areas. For example, the school vaccination program must be intensified and expanded to improve immunization coverage in urban areas. Similarly, our findings underscores the urgency of developing and upscaling user-friendly close-to-client health care options (parallel, but not similar, to CHPS) that can work in the more complex urban environment.

In addition, immunization campaigns should focus attention on women and children in higher socioeconomic households to address the emerging gaps in immunization coverage. Lastly, the findings highlight the importance of women empowerment, for example, through education and employment to child health outcomes. As such, child health interventions should be situated within broader strategies of women empowerment and decision making within the household through improving female education and employment. This paper amplifies recommendations that have also been identified in previous studies. Implementation, however, has been slow and may call for application of more diverse research methods for achieving an in-depth understanding of the factors promoting or hindering uptake of routine childhood immunization.

## Abbreviations

BCG: Bacillus Calmette-Guèrin
CHPS: Community-based Health Planning and Services
DPT: Diphtheria-Pertusis-Tetanus
EPI: Expanded Programme on Immunizations
GAVI: Global Alliance for Vaccines and Immunizations
GDHS: Ghana Demographic and Health Survey
GHS: Ghana Health Services
GSS: Ghana Statistical Service
MDGs: Millennium Development Goals
SDGs: Sustainable Development Goals
SSA: Sub-Saharan Africa
UNICEF: United Nations Children’s Emergency Fund
WHO: World Health Organisation
